# Synthesis and cytotoxic evaluation of some new[1,3]dioxolo[4,5-*g*]chromen-8-one derivatives

**DOI:** 10.1186/2008-2231-22-41

**Published:** 2014-05-02

**Authors:** Eskandar Alipour, Zinatsadat Mousavi, Zahra Safaei, Mahboobeh Pordeli, Maliheh Safavi, Loghman Firoozpour, Negar Mohammadhosseini, Mina Saeedi, Sussan Kabudanian Ardestani, Abbas Shafiee, Alireza Foroumadi

**Affiliations:** 1 Department of Chemistry, Islamic Azad University, Tehran-North Branch, Zafar St, Tehran, Iran; 2 Department of Biochemistry, Institute of Biochemistry and Biophysics, University of Tehran, Tehran, Iran; 3 Biotechnology Department, Iranian Research Organization for Science and Technology, Tehran, Iran; 4 Drug Design and Development Research Center, Tehran University of Medicinal Sciences, Tehran, Iran; 5 Department of Medicinal Chemistry, Faculty of Pharmacy and Pharmaceutical Sciences Research Center, Tehran University of Medical Sciences, Tehran, Iran

**Keywords:** Homoisoflavonoids, [1,3]dioxolo[4,5-*g*]chromen-8-one, Cancer, Cytotoxic activity

## Abstract

**Background:**

Homoisoflavonoids are naturally occurring compounds belong to flavonoid classes possessing various biological properties such as cytotoxicity. In this work, an efficient strategy for the synthesis of novel homoisoflavonoids, [1,3]dioxolo[4,5-*g*]chromen-8-ones, was developed and all compounds were evaluated for their cytotoxic activities on three breast cancer cell lines.

**Methods:**

Our synthetic route started from benzo[*d*][1,3]dioxol-5-ol which was reacted with 3-bromopropanoic acid followed by the reaction of oxalyl chloride to afford 6,7-dihydro-8*H*-[1,3]dioxolo[4,5-*g*]chromen-8-one. The aldol condensation of the later compound with aromatic aldehydes led to the formation of the title compounds. Five novel derivatives **4a-e** were tested for their cytotoxic activity against three human breast cancer cell lines including MCF-7, T47D, and MDA-MB-231 using the MTT assay.

**Results:**

Among the synthesized compounds, 7-benzylidene-6,7-dihydro-8*H*-[1,3]dioxolo[4,5-*g*]chromen-8-one (**4a**) exhibited the highest activity against three cell lines. Also the analysis of acridine orange/ethidium bromide staining results revealed that 7-benzylidene-6,7-dihydro-8*H*-[1,3]dioxolo[4,5-*g*]chromen-8-one (**4a**) and 7-(2-methoxybenzylidene)-6,7-dihydro-8*H*-[1,3]dioxolo[4,5-*g*]chromen-8-one (**4b**) induced apoptosis in T47D cell line.

**Conclusion:**

Finally, the effect of methoxy group on the cytotoxicity of compounds **4b-4d** was investigated in and it was revealed that it did not improve the activity of [1,3]dioxolo[4,5-*g*]chromen-8-ones against MCF-7, T47D, and MDA-MB-231.

## Background

Homoisoflavonoids, naturally occurring compounds belong to flavonoid classes and possess a wide spectrum of biological properties such as anti-inflammatory [1], antioxidant [2], antiproliferative [3], antifungal [4], antiviral [5], and antimutagenic activities [6]. They mainly include a chromanone, chromone, or chromane skeleton and are ubiquitous in plants such as *Ophiopogon*[7], *Polygonatum*[8], *Scilla*[9], *Eucomis*[10], and *Muscari*[11]. Recently, several homoisoflavonoids have been successfully isolated from plants and evaluated for their bioactivities [12,13].

Chalcones have been the center of attention owing to their significant biological activities [14-18]. Also they are the most important precursors for the formation of α, β-unsaturated carbonyl system in flavonoid classes. Homoisoflavonoids including chalcone system have shown selective biological activities [19]. The isolated natural homoisoflavonoids having 3-benzylidenechroman-4-one skeleton were found to be potent and selective MAO-B inhibitors. Compounds involving benzylidene chromanone have depicted significant medicinal properties such as antioxidant [20], anticancer [21], anti-inflammatory [22], anti-human-immune deficiency virus (HIV-I) activities [23].

**Figure 1:**
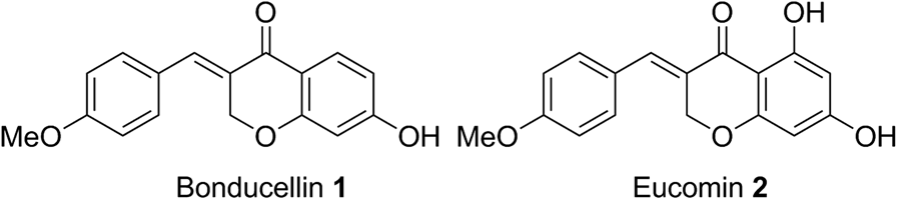
Bonducellin 1 and Eucomin 2.

Two naturally occurring homoisoflavonoids, bonducellin [24]**1** and eucomin [25]**2** (Figure 1), isolated from *Caesalpiniabonducella* and *Eucomis bicolor* BAK (Liliaceae) were considered. These compounds and their synthetic analogues have shown important biological properties such as anti-tuberculosis activity [26] and inhibition of protein tyrosine kinase (PTK) [27].

**Scheme 1:**
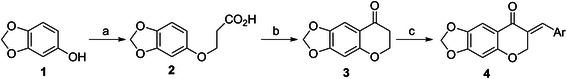
**Synthesis of [1,3]dioxolo[4,5-*g*]chromen-8-ones 4. (a) **NaOH, Na_2_CO_3_, Br(CH_2_)_2_COOH, H_2_O, reflux, **(b) **oxalyl chloride, SnCl_4_, benzene, **(c) **aromatic aldehydes, HCl (g), 0°C.

On the synthesis of bioactiveheterocycles containing oxygen specially chalcones and homoisoflavonoids [9,21,28,29]; herein, we focused on new substituted [1,3]dioxolo[4,5-*g*]chromen-8-one derivatives **4** to profit from both chalcones and homoisoflavonoids (Scheme 1). Then, we evaluated their cytotoxic activities against three human breast cancer cell lines; MCF-7, T47D, and MDA-MB-231 using the MTT assay.

## Methods

### Chemistry

All starting materials, reagents, and solvents were prepared from Merck (Germany). Melting points were determined on a Kofler hot stage apparatus (Vienna, Austria) and are uncorrected. ^1^H-NMR spectra were recorded using a Bruker 400 spectrometer (Bruker, Rheinstatten, Germany), and chemical shifts are expressed as δ (ppm) with tetramethylsilane (TMS) as internal standard. The IR spectra were obtained on a Nicolet Magna FT-IR 550 spectrophotometer (potassium bromide disks).

#### ***General procedure for the synthesis of [1,3]dioxolo[4,5-g]chromen-8-one derivatives 4***

3-(Benzo[*d*][1,3]dioxol-5-yloxy)propanoic acid **2** and 6,7-dihydro-8*H*-[1,3]dioxolo[4,5-*g*]chromen-8-one **3** prepared according to [30] (Scheme 1).

Dry hydrogen chloride gas was passed through an ice-cold solution of 6,7-dihydro-8*H*-[1,3]dioxolo[4,5-*g*]chromen-8-one **3** (0.5 mmol) and benzaldehyde derivative (0.7 mol) in absolute EtOH (3 mL) for 2 min. The reaction mixture was allowed to stand at room temperature for 48 h. The precipitated product was filtered off, dried, and recrystallized from ethanol and water.

#### ***7-Benzylidene-6,7-dihydro-8H-[1,3]dioxolo[4,5-g]chromen-8-one (4a)***

Yield: 48%, mp 141–144°C. IR (KBr): 1664 (C = O) cm^-1^. ^1^H-NMR (CDCl_3_, 400 MHz) δ: 7.83 (s, 1H, benzylidene), 7.43 (s, 1H, H_9_), 7.42-7.25 (m, 5H, Ph), 6.41 (s, 1H, H_4_), 6.00 (s, 2H, H_1_, CH_2_), 5.29 (s, 2H, CH_2_). Anal. Calcd. for C_17_H_12_O_4_: C, 72.85; H, 4.32. Found: C, 72.68; H, 4.18.

#### ***7-(2-Methoxybenzylidene)-6,7-dihydro-8H-[1,3]dioxolo[4,5-g]chromen-8-one (4b)***

Yield: 31%, mp 160–163°C. IR (KBr): 1660 (C = O) cm^-1^. ^1^H-NMR (CDCl_3_, 400 MHz) δ: 7.961 (s, 1H, benzylidene), 7.39 (s, 1H, H_9_), 7.04-6.94 (m, 4H, H_3′_, H_4′_, H_5′_, H_6′_), 6.40 (s, 1H, H_4_), 6.00 (s, 2H, H_1_, CH_2_), 5.17 (s, 2H, CH_2_), 3.86 (s, 3H, OCH_3_). Anal. Calcd. for C_18_H_14_O_5_: C, 69.67; H, 4.55. Found: C, 69.52; H, 4.41.

#### ***7-(3-Methoxybenzylidene)-6,7-dihydro-8H-[1,3]dioxolo[4,5-g]chromen-8-one (4c)***

Yield: 42%, mp 161–163°C. IR (KBr): 1662 (C = O) cm^-1^. ^1^H-NMR (CDCl_3_, 400 MHz) δ: 7.79 (s, 1H, benzylidene), 7.38 (s, 1H, H_9_), 7.04-6.93 (m, 4H, H_2′_, H_4′_, H_5′_, H_6′_), 6.41(s, 1H, H_4_), 6.00 (s, 2H, H_1_, CH_2_), 5.29 (s, 2H, CH_2_), 3.84 (s, 3H, OCH_3_). Anal. Calcd. for C_18_H_14_O_5_: C, 69.67; H, 4.55. Found: C, 69.83; H, 4.72.

#### ***7-(4-Methoxybenzylidene)-6,7-dihydro-8H-[1,3]dioxolo[4,5-g]chromen-8-one (4d)***

Yield: 31%, mp 169–172°C. IR (KBr): 1665 (C = O) cm^-1^. ^1^H-NMR (CDCl_3_, 400 MHz) δ: 7.79 (s, 1H, benzylidene), 7.38 (s, 1H, H_9_), 7.26 (d, *J* = 8.4 Hz, 2H, H_2΄_, H_6΄_), 6.96 (d, *J* = 8.4 Hz, 2H, H_3΄_, H_5΄_), 6.42 (s, 1H, H_4_), 6.00 (s, 2H, H_1_, CH_2_), 5.32 (s, 2H, CH_2_), 3.86 (s, 3H, OCH_3_). Anal. Calcd. for C_18_H_14_O_5_: C, 69.67; H, 4.55. Found: C, 69.53; H, 4.82.

#### ***7-(Benzo[d][1,3]dioxol-5-ylmethylene)-6,7-dihydro-8H-[1,3]dioxolo[4,5-g]chromen-8-one (4e)***

Yield: 42%, mp 198–200°C. IR (KBr): 1667 (C = O) cm^-1^. ^1^H-NMR (CDCl_3_, 400 MHz) δ: 7.73 (s, 1H, benzylidene), 7.37 (s, 1H, H_9_), 6.86-6.67 (m, 3H, H_3′_, H_4′_, H_6′_), 6.41 (s, 1H, H_4_), 6.03 (s, 2H, H_1′_,CH_2_), 6.00 (s, 2H, H_1_, CH_2_), 5.29 (s, 2H, CH_2_). Anal. Calcd. for C_18_H_12_O_6_: C, 66.67; H, 3.73. Found: C, 66.48; H, 3.55.

## Biological assay

### Cell lines and cell culture

Human breast cancer cell lines including MDA-MB231, MCF-7 and T47D cells were obtained from the National Cell Bank of Iran, Pasteur Institute, Tehran, Iran. Cancer cell lines were grown in RPMI-1640 medium supplemented with 10% heat-inactivated fetal calf serum, 100 μg/ml streptomycin and 100 U/ml penicillin at 37°C in a humidified atmosphere with 5% CO_2_.

#### ***In vitro cytotoxicity assay***

The in vitro cytotoxic activity of [1,3]dioxolo[4,5-*g*]chromen-8-ones **4a-e** was achieved using MTT colorimetric assay. The *in-vitro* cytotoxic activity of all synthesized compounds were evaluated against three human breast cancer cell lines including MCF-7, T47D and MDA-MB-231 using MTT colorimetric assay according to the method of Mosman [31]. Cancer cell lines were grown in RPMI-1640 medium supplemented with 10% heat-inactivated fetal calf serum (Gibco BRL), 100 μg/ml streptomycin and 100 U/ml penicillin at 37°C in a humidified atmosphere with 5% CO_2_.

Briefly, cultures in the exponential growth phase were trypsinized and diluted in complete growth medium to give a total cell count of 5 × 10^4^ cells/ml. 195 μl of the cell suspension was seeded into the wells of 96-well plates (Nunc, Denmark). The plates were incubated overnight in a humidified air atmosphere at 37°C with 5% CO_2_. After overnight incubation, 5 μl of the media containing various concentrations of the compounds was added per well in triplicate (final concentration 1, 5, 10 and 20 μg/ml). The plates were incubated for further 72 h. The final concentration of DMSO in the highest concentration of the applied compounds was 0.1%. In each plate, there were three control wells (cells without test compounds) and three blank wells (the medium with 0.1% DMSO) for cell viability. Etoposide and doxorubicine were used as positive controls for cytotoxicity. After treatment, the medium was removed and 200 μl phenol red-free medium containing MTT (1 mg/ml), was added to wells, followed by 4 h incubation. After incubation, the culture medium was then replaced with 100 μl of DMSO and the absorbance of each well was measured by using a microplate reader at 492 nm. For each compound, the concentration causing 50% cell growth inhibition (IC_50_) compared with the control was calculated from concentration response curves by regression analysis.

#### ***Fluorescence microscopy evaluation***

Acridine orange/ethidium bromide (AO/EB) double staining [32] was applied to observe the morphological changes in cell death induced by the most potent compounds **4a** and **4b**. Acridine orange is taken up by both viable and dead cells and emitting green fluorescence if intercalated into double stranded nucleic acid (DNA) or red fluorescence if bound to single stranded nucleic acid (RNA) due to its accumulation in lysosomes. Ethidium bromide is taken up only by cells with an altered cell membrane and emits red fluorescence by intercalation into DNA. Cells were seeded in 6-well plates (4 × 10^5^ cell/well) for 24 h. Then, cells were treated with IC_50_ concentration of test compounds for 24 h at 37°C with 5% CO_2_. After treatment, cells were washed twice with phosphate buffer saline (PBS) and then 1 μl of dye mixture (100 μg/ml AO and 100 μg/ml EB in PBS were mixed with 25 μl of cell suspension (0.4 × 10^6^ cells/well) on a clean microscope slide. The suspension was immediately examined by Axoscope 2 plus fluorescence micro- scope from Zeiss (Germany) at 40× magnification.

## Results and discussions

Benzo[*d*][1,3]dioxol-5-ol **1** (Scheme 1) was converted to 3-(benzo[d][1,3]dioxol-5-yloxy)propanoic acid **2** and subsequently to 6,7-dihydro-8*H*-[1,3]dioxolo[4,5-*g*]chromen-8-one **3** according to the procedure [30]. In the next step, we investigated the reaction of 6,7-dihydro-8*H*-[1,3]dioxolo[4,5-*g*]chromen-8-one **3** and 4-methoxybenzaldehyde to obtain the corresponding product, 7-(4-methoxybenzylidene)-6,7-dihydro-8*H*-[1,3]dioxolo[4,5-*g*]chromen-8-one (**4d**) (Table 1).

**Table 1:** **Chemical structures and *in vitro *** **cytotoxic activity (IC**_**50 **_**, μg/ml)**^**a **^**of compounds 4a-4e against breast cancer cell lines**

**Entry**	**Compound**	**MCF-7**	**T47D**	**MDA-MB-231**
1		6.2 ± 0.1	4.6 ± 0.1	9.3 ± 2.1
2		> 100	5.7 ± 0.07	27.3 ± 7.1
3		> 100	18.8 ± 2.3	29.05 ± 1.7
4		> 100	9.2 ± 2.9	> 100
5		> 100	> 100	> 100
6	Doxorubicin	0.002 ± 0.002	0.03 ± 0.002	0.006 ± 0.004
7	Etoposide	7.5 ± 0.32	7.9 ± 0.45	11.9 ± 0.87

^a^The IC_50_ values represent an average of three independent experiments (mean ± SD).

To run successful aldol condensation reaction, acid-catalyzed and base-catalyzed approaches were investigated using various conventional acids and base in different solvents. It was found that the aldol condensation was conducted in better yield in the presence of HCl (g).

Then, various derivatives including 7-(2-methoxybenzylidene)-6,7-dihydro-8*H*-[1,3]dioxolo[4,5-*g*]chromen-8-one (**4b**) and 7-(3-methoxybenzylidene)-6,7-dihydro-8*H*-[1,3]dioxolo[4,5-*g*]chromen-8-one (**4c**) possessing methoxy (OMe) group at ortho and meta positions were prepared to compare their bioactivities against the studied cell lines with that of the control. Also other two derivatives, 7-benzylidene-6,7-dihydro-8*H*-[1,3]dioxolo[4,5-*g*]chromen-8-one (**4a**) and 7-(benzo[*d*][1,3]dioxol-5-ylmethylene)-6,7-dihydro-8*H*-[1,3]dioxolo[4,5-*g*]chromen-8-one (**4e**) were prepared to investigate the effect of methxoy substituent on the cytotoxicity (Table 1).

The *in vitro* cytotoxic activity of [1,3]dioxolo[4,5-*g*]chromen-8-one derivatives **4**, were tested against three human breast cancer cell lines including MCF-7, T47D, and MDA-MB-231. The 50% growth inhibitory concentration (IC_50_) for all derivatives were calculated and depicted in Table 1.

According to MTT assay results in Table 1, 7-benzylidene-6,7-dihydro-8*H*-[1,3]dioxolo[4,5-*g*]chromen-8-one (**4a**) showed the highest activity against MCF-7, T47D, and MDA-MB-231 cell lines with IC_50_ values of 6.2 ± 0.1, 4.6 ± 0.1, and 9.3 ± 2.1 μg/ml, respectively. In contrast, 7-(benzo[*d*][1,3]dioxol-5-ylmethylene)-6,7-dihydro-8*H*-[1,3]dioxolo[4,5-*g*]chromen-8-one (**4e**) did not show any cytotoxicity at the concentrations used. It seems that the presence of benzo[*d*][1,3]dioxole in benzylidene moiety decreases the cytotoxic activity of the corresponding compound. As can be seen in Table 1 (Entries 2–4), by introduction of OMe into the ortho, meta or para positions of benzylidenemoiety (compounds **4b**, **4c**, and **4d**), different results were observed. All of them were inactive against MCF-7 cell line (IC_50_ > 100 μg/ml), whereas they exhibited good activity against T47D cell line with IC_50_ values of 5.7 ± 0.07, 18.8 ± 2.3, and 9.2 ± 2.9 μg/ml, respectively. It should be noted that compounds **4b** and **4c** were active against MDA-MB-231 cell line and **4d** did not show any activity in this cell line. Presence of OMe in benzylidene moiety did not play crucial role on the improvement of cytotoxicity effects.

**Figure 2:**
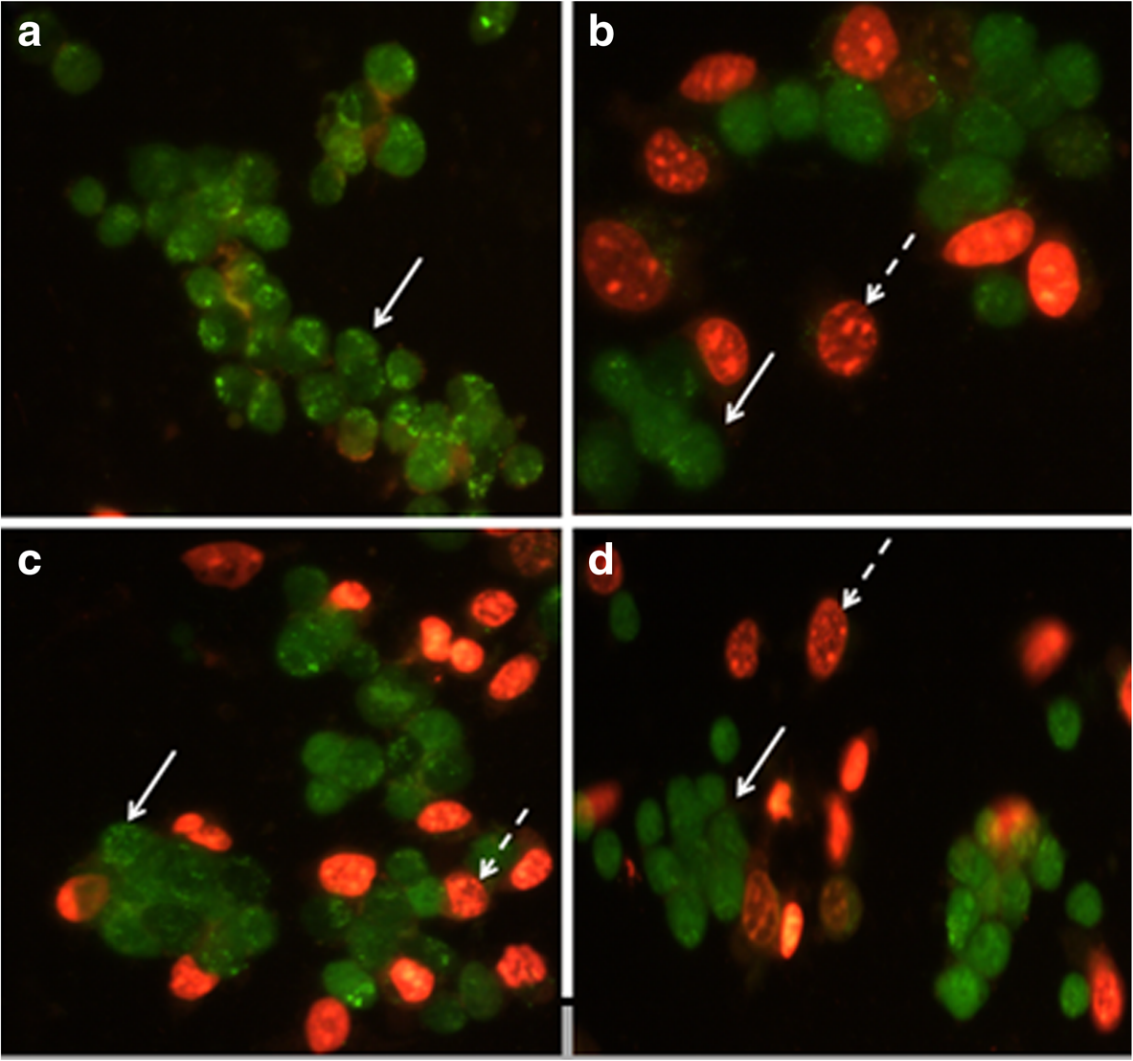
**Morphological analysis of T47D cells treated with 4a and 4b by acridine orange/ethidium bromide double staining method. a)** DMSO 1% as control, **b)** etoposide as positive control, **c)** cells treatedwith 4a for 24 h. **d)** cells treatedwith 4b for 24 h. White arrow indicates live cells, dashed arrow shows apoptotic cells. The images of cells were taken with a fluorescence microscope at 400 × magnification.

To study the effect of our synthetic compounds on cell lines, acridine orange/ethidium bromide double staining technique was used to evaluate the occurrence of apoptosis in cells. Analysis of the acridine orange/ethidium bromide staining results showed that 7-benzylidene-6,7-dihydro-8*H*-[1,3]dioxolo[4,5-*g*]chromen-8-one (**4a**) and 7-(2-methoxybenzylidene)-6,7-dihydro-8*H*-[1,3]dioxolo[4,5-*g*]chromen-8-one (**4b**) induced apoptosis in T47D cell line (Figure 2). The cells treated with the most potent compounds increased the extent of apoptosis relative to untreated control cells. As shown in Figure 2, the non-apoptotic control cells were stained green and the apoptotic cells had orange particles in their nuclei due to nuclear DNA fragmentation.

## Conclusion

In conclusion, novel [1,3]dioxolo[4,5-*g*]chromen-8-one derivatives were synthesized and tested for their cytotoxic activity against three human breast cancer cell lines including MCF-7, T47D, and MDA-MB-231 using the MTT assay. 7-Benzylidene-6,7-dihydro-8*H*-[1,3]dioxolo[4,5-*g*]chromen-8-one (**4a**) showed the highest activity against the three studied cell lines. Also the analysis of acridine orange/ethidium bromide staining results revealed that the cytotoxic effect of 7-benzylidene-6,7-dihydro-8*H*-[1,3]dioxolo[4,5-*g*]chromen-8-one (**4a**) and 7-(2-methoxybenzylidene)-6,7-dihydro-8H-[1,3]dioxolo[4,5-g]chromen-8-one (**4b**) may be due to inducing apoptosis in cancer cell lines.

## Competing interests

The authors declare that they have no competing interests.

## Authors’ contributions

EA: Supervision of the synthetic part. ZM: Synthesis of the title compounds. ZS: Synthesis of the title compounds. MP: Performed the cytotoxic tests. MS: Performed the cytotoxic tests and collaborated in manuscript preparation. LF: Design of target compounds. NM: Synthesis of the title compounds. MS: collaborated in manuscript preparation. SKA: Supervision of the cytotoxic tests. AS: Collaboration in identifying the structures of target compounds. AF: Design of target compounds and supervision of the synthetic and pharmacological parts. All authors read and approved the final manuscript.
